# Fabrication and Characterization of Steel-Base Metal Matrix Composites Reinforced by Yttria Nanoparticles through Friction Stir Processing

**DOI:** 10.3390/ma14247611

**Published:** 2021-12-10

**Authors:** Essam R. I. Mahmoud, Hamad Almohamadi, Abdulrahman Aljabri, Sohaib Z. Khan, Ahmad N. Saquib, Mohammed Farhan, Mohammed Abdel-Ghani Elkotb

**Affiliations:** 1 Department of Mechanical Engineering, Islamic University of Madinah, Medina 42351, Saudi Arabia; aaljabri@iu.edu.sa (A.A.); szkhan@iu.edu.sa (S.Z.K.); ansaquib@gmail.com (A.N.S.); contactfarhanium@gmail.com (M.F.); 2 Central Metallurgical Research and Development Institute (CMRDI), Cairo 11421, Egypt; 3 Department of Chemical Engineering, Islamic University of Madinah, Medina 42351, Saudi Arabia; hha@iu.edu.sa; 4 Mechanical Engineering Department, College of Engineering, King Khalid University, Abha 61421, Saudi Arabia; melkotb@kku.edu.sa; 5 Mechanical Engineering Department, Faculty of Engineering, Kafrelsheikh University, Kafr El Sheikh 33516, Egypt

**Keywords:** friction stir processing, steel-base metal matrix composites, yttrium oxide, microstructure, hardness, mechanical properties

## Abstract

Friction Stir Processing (FSP) was used to fabricate metal matrix composite, based on steel and reinforced with nano-sized yttrium oxide powder. The powder was packed in a narrow longitudinal groove of 2 mm depth and 1 mm width cut in the steel plate’s rear surface. Different rotation speeds of 500–1500 rpm were used, at a fixed traveling speed of 50 mm·min^−1^. Single-pass and two passes, with the same conditions, were applied. The direction of the second pass was opposite to that of the first pass. After the first pass, complete nugget zones were obtained when the rotation speeds were more than 700 rpm with some particles agglomeration. The added particles showed as narrow elliptical bands, with a band pitch equal to the rotation speed over traveling speed. Performing the second FSP pass in the opposite direction resulted in better particles distributions. Almost defect-free composite materials, with homogenously distributed yttria nano-sized particles, were obtained after two passes when rotation speeds more than 700 rpm were used. The resulting steel matrix grains were refined from ~60 μm of the base metal to less than 3 μm of the processed nugget zone matrix. The hardness and the tensile strength of the fabricated materials improved almost two-fold over the base metal. Uniform microhardness values within the nugget areas were observed at higher rotational speeds. The ductility and toughness of the fabricated composites were reduced compared to the base metal.

## 1. Introduction

Steels remain one of the most important alloys in our life. They are used as construction materials in a wide variety of applications: buildings, automobiles, machine components, chemical, oil and gas, defense, aerospace, marine, medicine, food processing, pressure vessels, and steam turbine rotor applications. This is due to the low cost, high melting point, thermal stability, and good toughness at relatively low and high temperatures [1,2]. However, steels’ relatively limited strength and low wear resistance have been considered insufficient in specific applications [3]. Metal Matrix Composites (MMCs), based on steels and reinforced with ceramic particles, show better material properties that cannot be obtained from other traditional alloys [4]. MMCs can improve the strength, stiffness, hardness, creep behavior, and wear resistance, making them promising candidates for more special applications or, at least, prolonging and enhancing their service life [5]. Comparing with non-ferrous alloys, steel-based MMCs are less common because steel has a high melting point. Mixing and dispersing insoluble ceramics, which have different densities, in liquid steel is not an easy process [6,7]. Several liquid state methods have been commonly used to fabricate MMCs, including casting [8], stir casting [9], infiltration process [10], and laser technology [11]. Non-homogeneity of the added particles, poor wettability, porosity, formation of undesirable phases, and pouring defects are the main drawback of these methods [12]. To overcome liquefaction problems, MMCs can be fabricated through solid-state processes or, in other words, below the matrix melting point. Solid-state processes will avoid undesirable reactions between the constituents, preventing the formation of brittle interdendritic and eutectic phases and the relative uniformity in the reinforcement distribution inside the matrix. It also provides microstructural refinements [13]. One of the recent techniques of solid-state composite fabrication is Friction Stir Processing (FSP). FSP is considered environment friendliness, less energy consumption, versatility, and no shielding gas is required. It is regarded as one of the most optimal, cost-effective, easy, and reliable processes for improving the microstructures of an existing alloy or even building new advanced alloys [14,15]. FSP, as a fabrication technique, has a non-consumable rotating shouldered probe, which is inserted into the top surface of the plate until it is heated and then traveled forward. Localized below melting point heating is generated in addition to severe plastic deformation in the processed zone [16]. The material during FSP, which is flowing around the tool pin and under the high pressure of the tool shoulder, making it the most suitable method to distribute particles inside the matrix [17]. The processed zone is constituted by recrystallized fine grains [18,19]. FSP probe wear is one of the most serious issues in composite fabrication, due to the hard particles that erode the tool surface during rotation, especially for relatively high temperatures achieved while using steel as the matrix [20]. To overcome the tool erosion issue, some hard probe materials, such as poly cubic boron nitride and tungsten carbide, are successfully employed in many works [20,21,22]. Another important technical aspect in the fabrication of composites through FSP is the way of introducing or packing the ceramic powder before the FSP. Most of the previous works filled the powder in a small groove cut in the upper surface. However, the powder will be expelled out by the action of the rotating tilted tool. To solve this problem, some works suggested that the powder filled in a groove cut on the top surface of the substrate, and it can be covered with a thin sheet of the same matrix materials [23,24]. In this case, the thin cover sheet cannot be enough to withstand the shear forces, by the rotating tool, to be broken and removed from the processed area. Other researchers suggested closing the powder-filled-groove with a pinless tool, then processed the composites with the normal tool with a pin [25,26]. Some of the packed powders can be expelled out during the pass by the tilted pinless tool shoulder.

Although many typical ceramics, such as SiC, WC, Al_2_O_3_, and TiC, are used as reinforcement in the fabrication of MMCs for strength improvement, corrosion, and wear resistance applications, Yttrium oxide (Yttria) can also be a possible candidate to be used as a proper reinforcement phase [27]. Yttria possesses high hardness, high refractive index, low-thermal expansion, high thermal stability, and high melting temperature. For these properties, yttria is one of the most optimum refractories for creep resistance applications. It will not only refine the matrix grains but also stabilize the microstructure when it is exposed to high temperatures [28,29]. There are several studies that used yttria as a reinforcement phase in the fabrication of aluminum-based composites. Bouaeshi et al. [30] studied the mechanical effect of a Y_2_O_3_ addition to aluminum, using an arc melting process. It was concluded that the microstructure of aluminum became more refined when the added yttria was increased. These increments have also contributed to the improvement in the wear resistance in the fabricated composites. Gwang-Ho Kim et al. [31] studied the dispersion behavior of Y_2_O_3_ in aluminum alloy and its effects on mechanical properties and microstructure as a function of oxide concentration. They reported uniform dispersion behavior of oxide particles at 2 wt.%. On the contrary, at 3 wt.%, reinforced composite had particle aggregation, and it was concluded that hardness increased by 20%, tensile strength increased by 55% compared with pure aluminum at 2 mass% oxides. J Ramesh Kumar et al. [32] successfully fabricated aluminum AAA6082/Y_2_O_3_ surface composites using FSP. They concluded that the oxide particles were distributed uniformly through the friction stirred zone. The hardness of the composite raised by 60% compared with the base metal. Besides, the wear characteristics of the composite also depended on the volume percentage of the oxide additive.

In literature, there are few works dealing with steel-based composites, reinforced with different ceramics, through FSP. From the previous open literature studies, the distribution of yttrium oxide as a reinforcement phase inside the steel matrix by FSP has never been reported. Therefore, the present work aims to develop new composite materials based on steel, as a matrix and reinforced with yttrium oxide nano-sized particles, through FSP with different rotational speeds. To solve the problem of power-spelling-out during the processing, the powder is added in a novel way on the rare side of the plate, opposite to the tool. In this case, the powder-filled in the groove was pressurized with the backing plate, leaving no extra space and maximum powder utilization. The macro/microstructures, of the developed MMCs through FSP, are investigated in detail, correlated with the hardness measurements around the vicinity of the nugget and the matrix. Moreover, tensile strength and impact toughness are investigated at room temperature.

## 2. Materials and Methods

**Figure 1:**
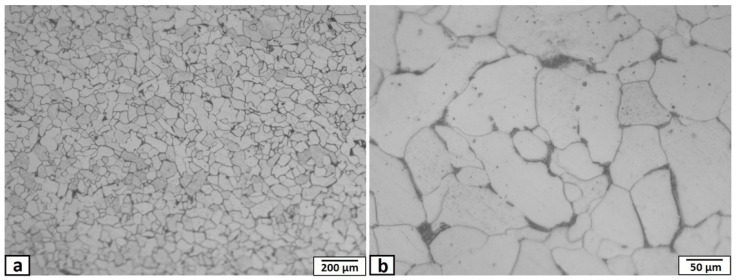
Optical micrograph of the mild steel matrix microstructure (**a**), and (**b**) enlarged image of (**a**).

**Figure 2:**
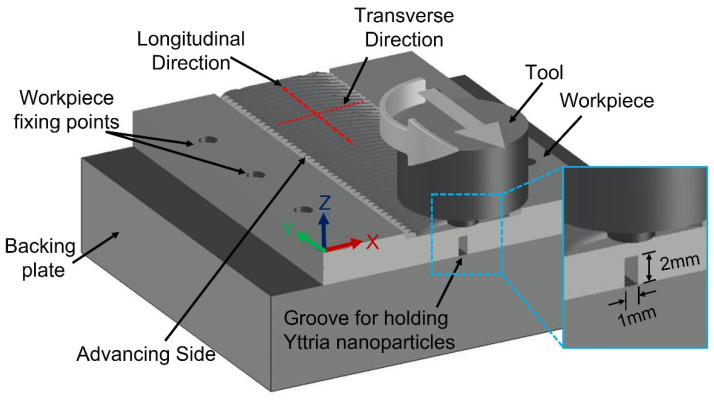
Schematic diagram of the FSP showing the groove for packing of yttria nano-sized particles relative to the tool and fixture.

Plates of mild steel, 3 mm thick, were used as a matrix with chemical composition and hardness listed in Table 1. It consisted mainly of equiaxed grains of ferrite structure with a small amount of pearlite, due to the low percentage of carbon, with an average grain size of ~60 μm, as shown in Figure 1. The reinforcement particles were nano-sized yttrium oxide (purity of 99.9%) with an average particle size of 40 nm. The FSP-composite was performed in this study using a vertical type milling machine with a power of 9 kW. The ready-to-use tool was fabricated from tungsten carbide powder through the powder metallurgy technique. Tool dimensions consisted of a shoulder of 15 mm diameter with a recessed angle of 10 degrees, a 6 mm diameter probe, and 2.9 mm length. The tool was used at a tilt angle of 3°. The yttrium oxide powder was packed in a narrow longitudinal groove of 2 mm depth and 1 mm width cut in the steel plate’s rear surface, on the opposite to the FSP tool, as shown in the schematic view in Figure 2. The narrow vertical groove helped in maintaining the packed power in its position during handling, sample fixation on the machine bed, and during the FSP operations. Besides avoiding powder dispersion in the air, the groove position ensured the powders had direct contact with approximately two-thirds the length of the tool pin during processing. The FSP tool was located and traveled precisely at the center of the groove. The FSP tool was rotated at rotation speeds ranging from 500–1500 rpm, at a fixed traveling speed of 50 mm·min^−1^. Single-pass and two passes, with the same conditions, were applied. The direction of the second pass was opposite to that of the first pass. After the FSP, the nugget zones were sectioned along with the traverse to the traveling direction and longitudinally for metallographic examinations. The macro/microstructures of the FSP nugget zone were investigated, using an optical microscope (Olympus optical microscope with digital camera, Madinah, Saudi Arabia) and scanning electron microscope (Philips XL30 ESEM environmental SEM) SEMTech Solutions, North Billerica, MA, USA) equipped with Oxford Instruments INCA 250 EDX system analyzer, after standard methods of metallography, and immersing the samples in an etchant of 2% Nital (nitric acid and ethyl alcohol).

**Figure 3:**
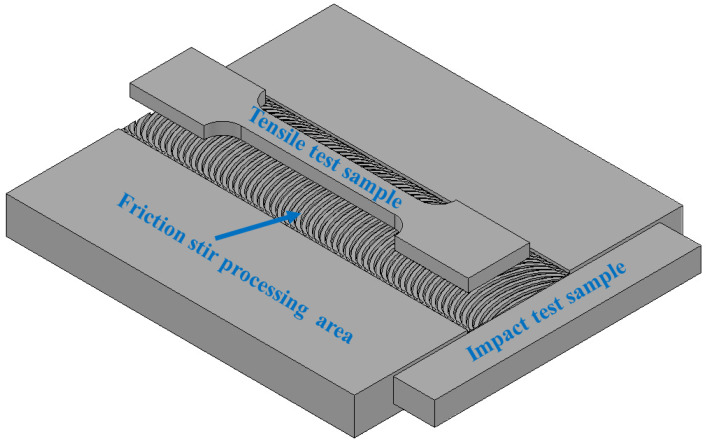
Schematic view of the location of tensile and impact samples.

The microhardness of the nugget zone was measured with a Vickers hardness tester. The hardness distribution was measured along a transverse horizontal line in the nugget zone that was 1.5 mm deep from the top surface. The tensile test was conducted on the FSPed composite samples after the second pass through the universal tensile testing machine, with a capacity of 100 kN and a strain rate of 5 mm/s. The samples were taken from the FSP zone center through the traveling direction with width and depth (thickness) of 5 mm and 2 mm, as shown in Figure 3. Moreover, the toughness of the processed samples was evaluated at room temperature through the Charpy impact test (Pendulum impact testing machine, Instron, Norwood, MA, USA) on unnotched samples with dimensions of 2.5 mm × 10 mm × 55 mm. The test was conducted three times for each condition, and the average was used in the evaluation.

## 3. Results and Discussion

### 3.1. General Features of the Stir Zone

**Figure 4:**
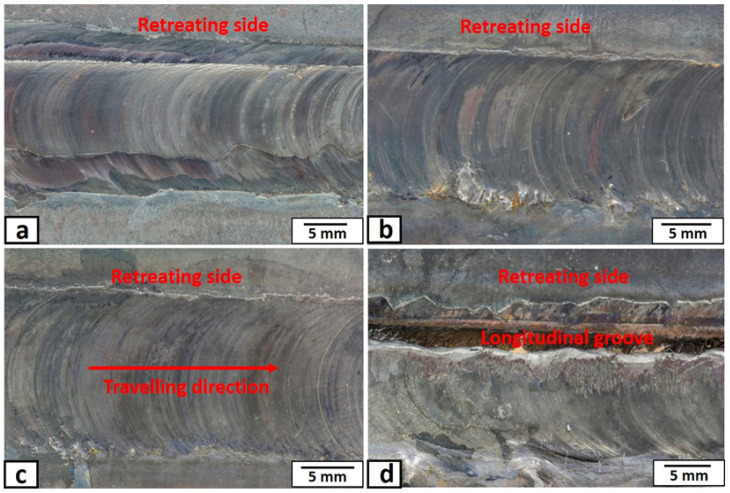
Optical micrographs showing the top view of the FSP nugget zones surface, fabricated by single FSP pass at tool rotational speed of (**a**) 1500 rpm, (**b**) 1000 rpm, (**c**) 700 rpm, and (**d**) 500 rpm.

**Figure 5:**
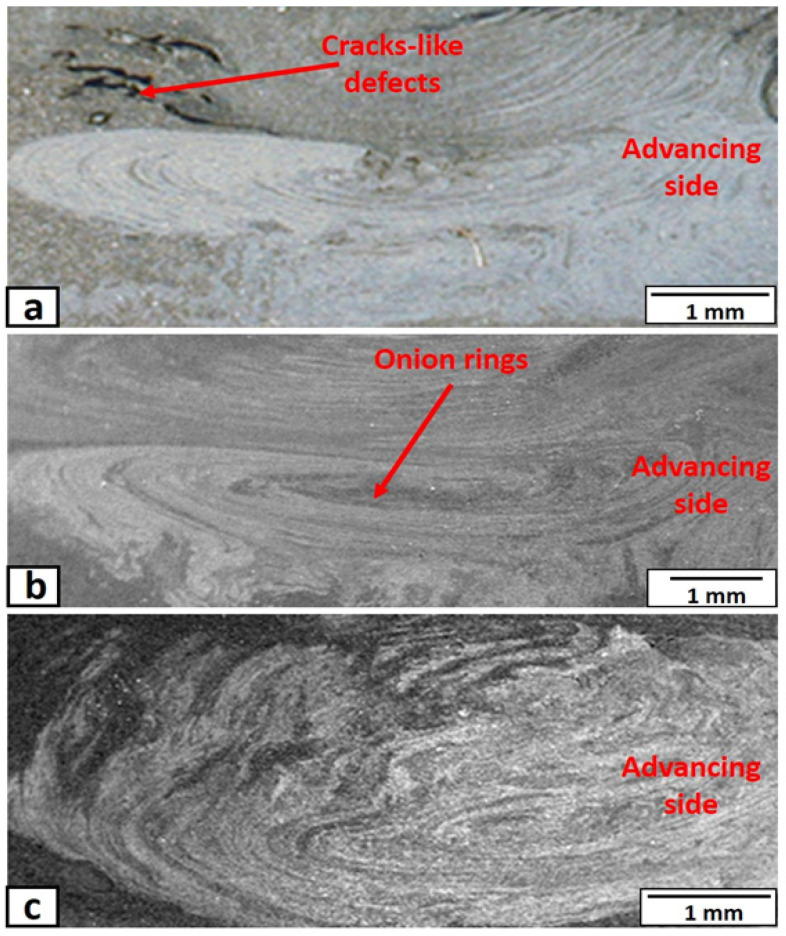
Macrographs of the nugget zone transverse cross-sections, fabricated by FSP single pass at tool rotational speed of (**a**) 700 rpm, (**b**) 1000 rpm, and (**c**) 1500 rpm.

The top macroscopic appearances of the nugget zone, produced at different rotational speeds, are shown in Figure 4. The top surfaces of the nugget zones, produced by a rotation speed of more than 500 rpm, were almost free of macro-defects as shown in Figure 4a–c. At the rotation speed of 500 rpm, the tool acted as a driller and made a longitudinal groove on the retreating side, as shown in Figure 4d. This may be due to the insufficient frictional heat, materials flow, and forging pressure generated by the tool at this low rotation speed, which left a tunnel of continuous cavities in the retreating side (the direction of the tool rotation is opposite to that of the tool traveling) of nugget zone. Moreover, the friction stir processed zone’s surface appeared as “C” shape towards the tool traveling direction in all rotation speeds. The C-shape was a typical result of the tool tilt angle (3°) and its rotation, which transferred the material from the tool’s front to the back, with pressure by the tool shoulder. In addition, the width of the nugget zones in all processed conditions was approximately equal to the shoulder diameter (15 mm). The tool shoulder serves as the main source of heat generation, and it controls the volume of the plasticized materials in the transverse direction (nugget width) [33]. Macroscopic appearances of the transverse-cross-sections (X-Z plane) of the nugget zones, produced by FSP single pass at different rotation speeds, are shown in Figure 5. Generally, the distribution of the added particles inside the nugget zone was unsymmetrical. Most of the yttrium oxide particles were distributed in the advancing side (the direction of the tool rotation is in the same direction of the tool traveling) and the nugget centers. This may be due to the retreating side’s materials undergoing less heat and plastic deformation because of the less frictional forces. The tool tilt angle of 3° increases the forging pressure at the tool’s backside, pressuring the materials to the advancing side. On the other hand, the front side of the tool undergoes less pressure (due to the tool tilt) and less heat generation. Thus, the materials transport to the retreating side with less pressure. The same observation was reported in the work of Avila et al. [34]. At the same time, at a lower rotation speed of 700 rpm, many longitudinal crack-like defects appeared in the upper part of the nugget zone, especially at the retreating side, as shown by the arrow in Figure 5a.

Such an area suffered from the tribble effects of low plastic deformation, generated by lower rotation speed, lower heat generation, and lower pressure. This resulted in loose materials layers with some cavities or cracks between them. The distribution of the added powder was improved by increasing the rotation speed without any serious defects such as cavities and cracks. This is due to the severe stirring action and more frictional heat generated at higher rotation speed (1000 rpm and more), which was sufficient to increase the temperature of the material to the suitable range, resulting in the strong plastic flow around the tool and form sound zones. From Figure 5, it is also noticed that the added yttrium oxide particles were distributed in the center of the nugget zones as squeezed onion ring features, especially at lower rotation speeds.

**Figure 6:**
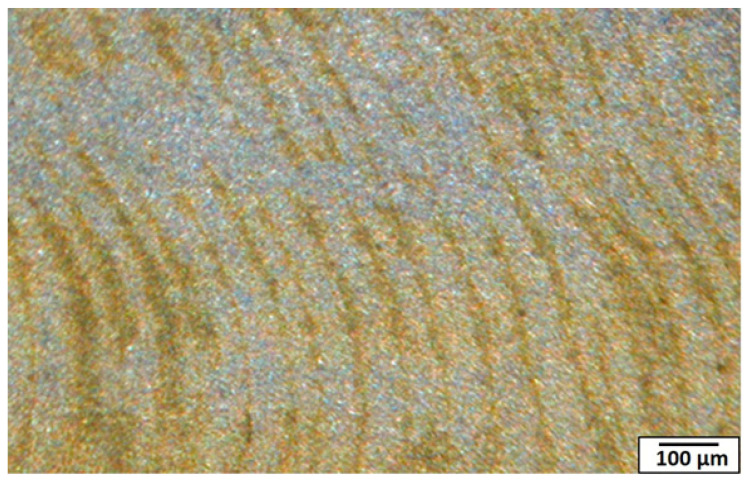
Macrograph of the nugget zone longitudinal cross-section, fabricated by FSP single pass at tool rotational speed of 700 rpm.

On the other hand, the yttrium oxide particles appeared in the longitudinal direction of the nugget zone cross-sections (Y-Z plane) as narrow elliptical bands, as shown in Figure 6. During the process, the pin pushed the materials upward in the front side, pressurized or forged by the shoulder in the backside, forming bands [35]. This material’s flow is repeated periodically, forming the same band pitch as the tool rotated and moved forward, as clearly shown in Figure 6. At rotation speed of 700 rpm, the band’s pitch was 68 µm, while it was 51 µm and 32 µm for rotation speeds of 1000 rpm and 1500 rpm, respectively. The pitch of these bands had a direct relationship with the rotation speed and traveling speed. By dividing the rotation speed over the traveling speed, almost the same values are obtained. For example, for the rotational speed of 700 rpm, it gives 14 rounds/mm (the traveling speed was fixed at 50 mm·min^−1^). Thus, the band pitch is 1/14 means 70 µm (close to the measured value of 68 µm). On the other hand, the FSP tool wear was found to be a severe problem. After processing a length of 500 mm at 700 rpm and 300 mm at 1500 rpm, the tool pin has almost vanished.

### 3.2. Microscopic Investigation of the Stir Zones after the First Pass

**Figure 7:**
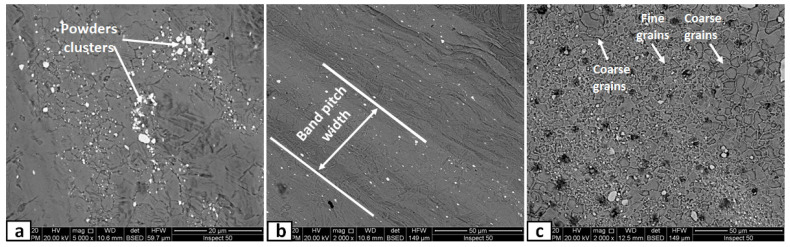
SEM micrographs of the nugget zone cross-section, fabricated by FSP single pass at tool rotational speed of (**a**) 700 rpm, (**b**) 1000 rpm, and (**c**) 1500 rpm.

**Figure 8:**
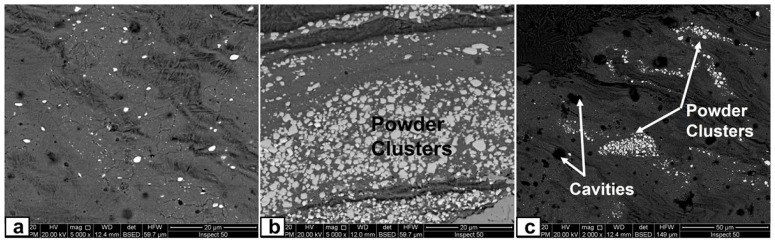
SEM micrographs of the nugget zone cross-section, fabricated by FSP single pass at tool rotational speed of 700 rpm at (**a**) advancing side, (**b**) nugget center, and (**c**) retreating side.

The microscopic appearances of the nugget zone, produced after a single FSP pass at different rotational speeds, are shown in Figure 7. At a lower rotation speed of 700 rpm, the yttrium oxide nano-sized particles (white color) were clustered in some batches, as shown in Figure 7a. The stirring action and the plastic deformation generated by the FSP tool were not enough to distribute the added particles throughout the nugget area. The distribution of the yttrium oxide nano-sized particles was improved by increasing the rotational speed. The added yttrium oxide was distributed homogeneously, inside the nugget zone in bands, by the FSP tool at this higher rotational speed. There was a clear difference in the banded area’s matrix grain size with yttrium oxide nano-sized particles and the adjacent area without nano-sized particles. The areas with powders had much more refined grains than those without powders. This is due to the pinning effect that will be discussed in Section 3.3. On the other hand, it is important to note that the yttrium oxide nano-sized particles were distributed in parallel bands, as demonstrated in Figure 7b. The yttrium oxide nano-sized particles were distributed in wide bands, leaving narrow bands with low particle density or even depleted from particles. The band pitch was 51 µm (Figure 7b) at a rotational speed of 1000 rpm. When the rotation speed was decreased to 500 rpm, many yttrium oxide nano-sized particles agglomeration and clusters were found in different areas in the nugget zone, as shown in Figure 8, especially in the lower part of the nugget zone, where the added yttrium oxide nano-sized particles were initially filled. Moreover, some holes (black color) were also detected, as shown by arrows in Figure 8c.

**Figure 9:**
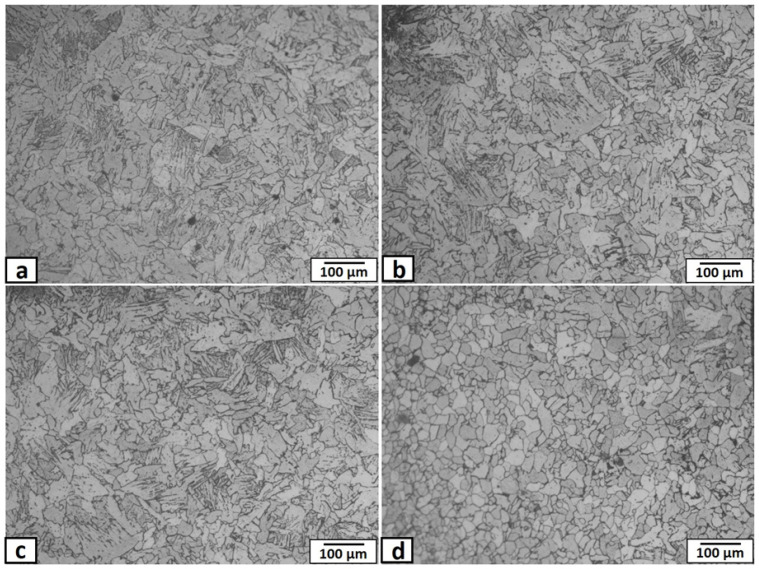
Optical micrographs of the heat-affected zone (HAZ) adjacent to the nugget zone, fabricated by FSP single pass at tool rotational speed of (**a**) 1500 rpm, advancing side, (**b**) 1000 rpm, advancing side, (**c**) 700 rpm, advancing side, and (**d**) 700 rpm, retreating side.

Regarding the heat-affected zones (HAZ), there is no significant difference between the HAZs beside the advancing side of nugget zones, produced by different rotational speeds, as shown in Figure 9a–c. Bainite and ferrite structure was obtained in all conditions. This is probably because the FSP is a cold working process with minimal heat generation, and it may also be due to the lower carbon content and other alloying elements in the mild steel substrate. For comparison, the HAZ in the retreating side (Figure 9d) for the 700 rpm condition showed mainly ferrite grains with a much lower amount of bainite. This may be due to the much lower heat generation in the retreating side with this lower rotational speed, which did not affect the adjacent base metal.

### 3.3. Microscopic Investigation of the Stir Zones after Second Passes

**Figure 10:**
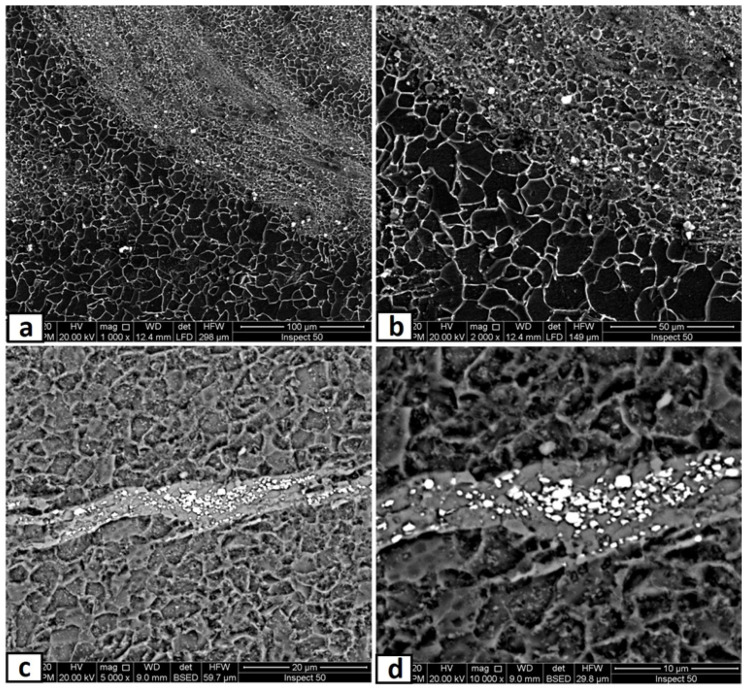
SEM micrographs at different magnifications of the nugget zone cross-section, fabricated by FSP double passes at tool rotational speed of 700 rpm at (**a**,**b**) advancing side, and (**c**,**d**) nugget center.

**Figure 11:**
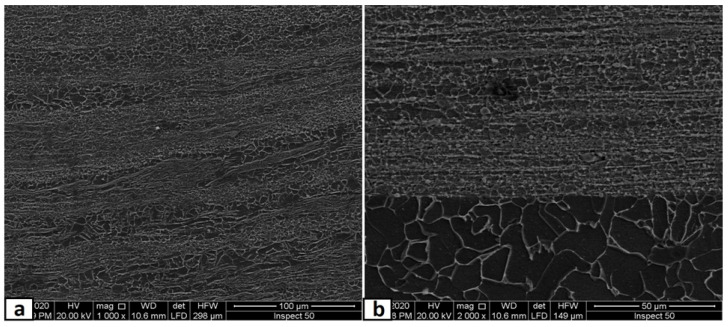
SEM micrographs at different magnifications of the nugget zone cross-section, fabricated by FSP double passes at tool rotational speed of 1000 rpm at (**a**) nugget center, and (**b**) advancing side.

**Figure 12:**
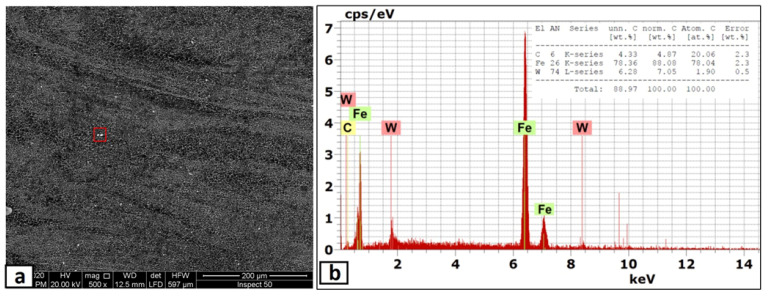
(**a**) SEM micrograph of the nugget zone cross-section, fabricated by FSP double passes at tool rotational speed of 1000 rpm, and (**b**) the EDX analysis of the marked area in (**a**).

In this part, the second FSP pass was applied on the first pass processed sample with the same condition. As mentioned in the experimental procedures, the direction of this second pass was the opposite of that of the first pass, which means the advancing side of the first pass was the second pass’s retreating side. This helped a lot in powder homogeneity and eliminated the unsymmetrical powder distributions in the nugget zone. The micrographs of the nugget zone, produced after double passes by the rotational speed of 700 rpm, are shown in Figure 10. The yttrium oxide nano-sized particles were distributed in fine ferrite grains on the advanced side (Figure 10a,b) and the nugget center (Figure 10c,d), forming a composite material. The grain refinement of the nugget zone can be noted compared with those of the adjacent thermo-mechanical affected zone. The ultra-fine grains of the steel matrix of fabricated composite materials reflected the severe plastic deformation and the dynamic recrystallization process by the FSP tool on one side. On the other side, the nano-sized yttrium oxide particles contributed to decreasing the steel matrix grain size, as it is precipitated as a secondary phase inside the matrix and created a pinning effect (called Zener pinning) to the steel grains. The precipitated particles, which are uniformly distributed in the steel matrix, act as obstacles in the grain boundaries, which stabilize the grain size and limit the grain growth during recrystallization [36]. On the other hand, some yttrium oxide nano-sized particles were clustered in a banded shape in the lower part of the nugget center, as shown in Figure 10c,d. This is due to the lower mixing action generated by the lower rotation speed. To avoid these clustering, the rotation speed was increased to 1000 rpm, and the results are shown in Figure 11. The yttrium oxide nano-sized particles were distributed as wide bands in the mild steel ferrite matrix without any noticeable defects in most nugget zones (Figure 11a). The low particle density areas became narrow. No clusters or agglomerations were observed. The FSPed zone interface with the adjacent base metal was clean and free from any defects, as shown in the magnified SEM image in Figure 11b. Some foreign particles were observed inside the nugget zone, which was analyzed by the EDX. The foreign particles were identified to contain tungsten as one of their constituents (Figure 12). It may be contaminated debris coming from the tool wear through the severe friction with the hard reinforcement and the matrix. The temperature and stresses generated during the severe plastic deformation of steel-matrix composite, reinforced with ceramic particles, are large enough to erode the tool pin [37].

**Figure 13:**
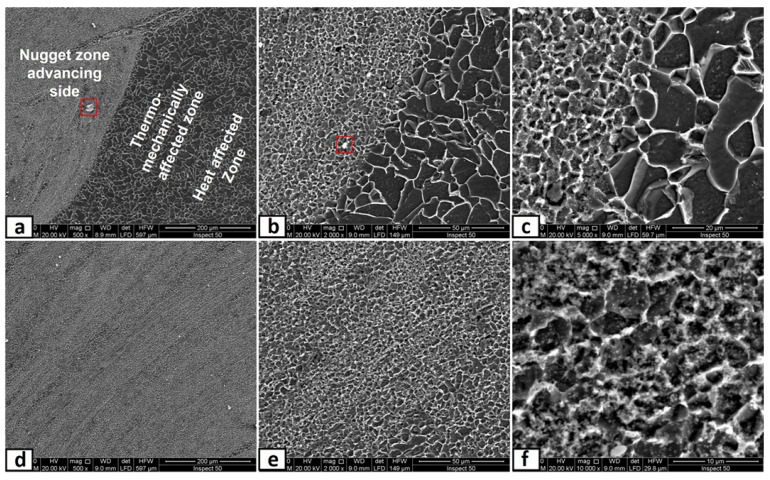
SEM micrographs at different magnifications of the nugget zone cross-section, fabricated by FSP double passes at tool rotational speed of 1500 rpm at (**a**–**c**) advancing side and (**d**–**f**) nugget center.

**Figure 14:**
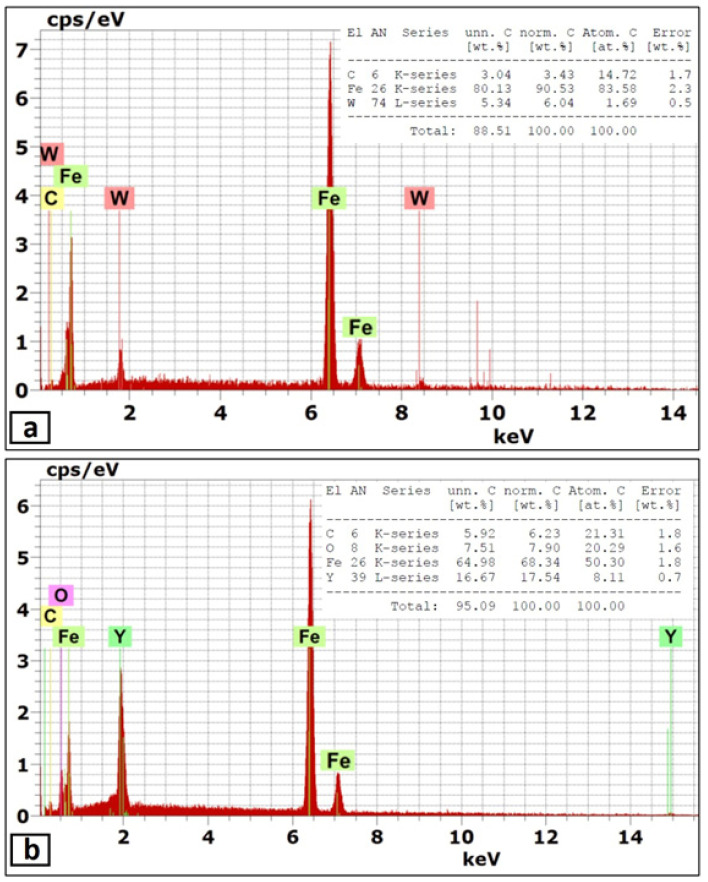
EDX analysis (**a**) of the marked area in Figure 12a and (**b**) of the marked area in Figure 12b.

When the rotation speed was increased to 1500 rpm, the homogenous distribution of yttrium oxide nano-sized particles, inside a ferrite matrix within the nugget zone, was achieved without any observable defect, as shown in Figure 13. The higher rotational speed even eliminates the particles’ banded structures, as clearly shown in Figure 13d,e. The observed results generally showed isotropic structure in the nugget zone, which is one of the unique merits of the FSP [38]. Another remarkable outcome was forming the clean and sound interface between the nugget zone and adjacent steel substrate, as shown in the magnified SEM image in Figure 13b,c. Regarding the grain size of the matrix of the nugget zone, it refined from ~60 μm (of the steel substrate) to lower than 1–3 μm by the double effect of the severe plastic deformation of the FSP and by the pinning effect of the added yttrium oxide nano-sized particles. The severe plastic deformation induced dynamic recrystallization, which refined the grains, and the pinning effect impeded the grain coursing. Regarding the relatively few large particles observed inside the nugget, the EDX analysis sometimes showed it as tungsten carbide (W element, Figure 14a) and yttrium oxide particles (Y and O elements, Figure 14b). For yttrium oxide, the nano-sized particles tend to be agglomerated by nature, in spite of the severe stirring action by the FSP tool shoulder and pin. On the other hand, the higher rotational speed accelerated the deterioration of the hard tool and the wear debris distributed inside the nugget zone.

### 3.4. Hardness Measurements

**Figure 15:**
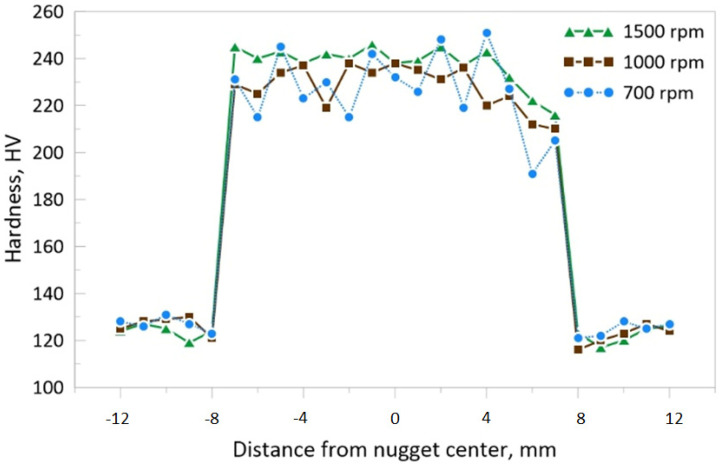
Hardness values measured at 1.5 mm deep from the upper surface of the nugget zone cross-section, fabricated by FSP double passes at different tool rotational speeds.

The hardness profiles in the nugget cross-section, produced after two passes at different rotational speeds, are shown in Figure 15. The measurements were carried out along a line approximately at a middle thickness of the substrate (1.5 mm from the upper edge). Generally, as compared with the base metal hardness of about 124 HV, the average hardness of the FSP nugget zone, with the addition of yttrium oxide nano-sized particles, was almost twice in all rotation speeds. This is due to the presence of the hard nano-yttrium oxide particles that were distributed inside the ferrite matrix. The grain refinement contributed significantly to this hardness increment. The hardness profiles reflected the homogeneity of the yttrium oxide nano-sized particles inside the nugget zone. For that reason, the hardness profile of the lower rotation speed (700 rpm) showed oscillating values. This is due to the particles that were distributed in narrow bands inside the nugget zone. The band that had a higher density of yttrium oxide nano-sized particles showed a higher hardness value, while the adjacent band that had a lower density of yttrium oxide showed relatively lower hardness values. In contrast, at a higher rotation speed of 1500 rpm, a relative uniformity in the hardness values homogeneity was recorded in the nugget zone. Similarly, the hardness values in the retreating side were lower than that of the advancing side and nugget center. These observations confirmed with the yttrium oxide nano-sized particles were uniformly and homogeneously distributed even below the top surface in the nugget zone.

### 3.5. Mechanical Properties

**Figure 16:**
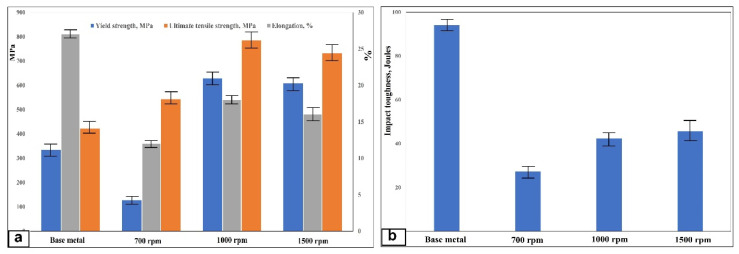
Tensile test results (**a**) and Charpy impact toughness measurements (**b**) of the fabricated composite at different rotation speeds, together with the base metal.

A remarkable improvement in tensile strength properties (yield strength and ultimate tensile strength) is observed for the steel/nano-Yttria composite materials, fabricated with two passes of FSP of a rotation speed of 1000 and 1500 rpm, which is almost two times that of the base metal, as shown in Figure 16a. This is due to many combined reasons. The first reason is the existence of nanoparticles inside the matrix, which will generate more dislocations around them, which needs more stresses to precede the plastic deformation. This can be considered as the work hardening, which enhances the tensile properties of the composites [39,40,41]. The nano-sized yttrium oxide particles that are dispersed in the matrix can change the direction crack propagation as it acts as crack bridging and increases the yield strength as more energy is required. Additionally, it can restrict the dislocation motion and deformation during loading, which leads to a significant reduction in elongation [42,43]. Secondly, during tensile loading, the load will be transferred from the ductile matrix to the stronger homogenous distributed nano-Yttria particles. Thirdly, the nano-Yttria particles will act as obstacles for the dislocation movement. Hence, it helps in increasing the tensile strength values [44]. Moreover, the effect of the grain refinement of the matrix shares in the strengthening effect (Hall–Petch relation) [45,46,47]. The sample, with a rotation speed of 1000 rpm, gives high tensile strength due to the matrix’s more ultra-fine grain structure. Nevertheless, with a further reduction in rotation speed to 700 rpm, a dramatic decrease in the tensile strength properties is recorded. This is because of the Yttria particle clusters that appeared, as discussed in the microstructure section. On the other hand, the results indicated that the elongation percentage is reduced for all the composite materials than for the base metal.

### 3.6. Impact Toughness

The impact toughness values of the composite materials, fabricated with two passes of different friction stir processing powers, were relatively lower than that of the low carbon steel base metal without yttrium oxide particles. Crack nucleation and propagation over the metallic single-phase metals consume more energy, as plastic deformation takes place before fracture. Moreover, the hard reinforcements in the composites act as stress concentrators that increase the local stress intensity in the composite and promote easy crack nucleation in the specimen. This is because the yield strength of the reinforcement and the matrix is different. Therefore, microvoids are easily nucleated at the boundaries between them. As seen in Figure 16b, very close impact toughness values were obtained at rotation speeds of 1000 and 1500 rpm. The toughness was in the range of 42–45 J, with a small standard deviation, since both rotation speeds show almost the same homogenous nano-sized yttrium oxide particles distributed in dynamic recrystallized ultra-fine grains structures. These values are much higher compared with other composites fabricated with other processes [48,49,50,51]. The matrix and reinforcement phases’ fine size delayed the crack nucleation and propagation since it seems to be a one-phase material. However, the lower rotation speed of 700 rpm samples showed a tendency for lower toughness (27 J). The yttrium oxide nano-sized particle clusters in the nugget lower part may have acted as stress concentration sites and preferential sites for crack nucleation and propagation.

## 4. Conclusions

The present work aimed to fabricate steel-base-metal matrix composites, reinforced with yttria nano-sized particles, through friction stir processing, using a tungsten-carbide tool. Yttrium oxide nano-sized particles were filled in a narrow groove, cut on the 3 mm thick mild steel plate’s rear side. Single-pass and double passes FSP, with different rotation speeds ranging from 500–1500 rpm, were applied. The nano-sized particle distributions and microstructure of the FSPed areas were investigated, together, with other mechanical behavior. The following conclusions can be obtained:

- Metal matrix composite composed of steel matrix and reinforced with yttria nano-sized particles was successfully fabricated through friction stir processing, at rotational speeds of more than 700 rpm. At a rotation speed of 500 rpm, the tool acted as a driller and destroyed the processed zone.
- After the first pass, the distributions of the yttria nano-sized particles were concentrated on the advancing side and the nugget centers. Some defect-like cracks appeared on the retreating side, especially at a lower rotation speed.
- The yttria nano-sized particles were distributed as narrow elliptical bands, and their band pitches were equal to the rotation speed (rpm) over traveling speed (mm/min).
- The application of the second pass, in the opposite direction of the first pass, improved the dispersion of the yttria nano-sized particles in the nugget zone, especially at higher rotation speeds. The added particles were distributed homogeneously throughout the nugget zone without any noticeable defects at the rotational speed of 1500 rpm.
- The grain size of the steel matrix was reduced to less than 2 μm after the application of double FSP passes by the effect of FSP severe plastic deformation and the pinning effect of the added particles. There was very narrow heat affect zone.
- After two passes, the hardness values of the FSPed zones were approximately twice that of the steel base metal in all the used rotational speeds. The distributions of the hardness values within the nugget zone were uniform, especially at higher rotation speeds.
- The fabricated composites’ tensile strength was significantly improved compared to the base metal, especially at a rotation speed of more than 700 rpm, while the results showed a reduction in elongation.
- The Charpy impact toughness values of the fabricated composites were almost half that of the base metal.

## Figures and Tables

**Table 1:** Chemical compositions and hardness of the base metal, wt.%.

Chemical Compositions (Wt. %)						Hardness, HV0.2N
C	Si	Mn	P	S	Fe	
0.08	0.26	0.33	0.02	0.01	Bal.	124

## Data Availability

Not applicable.
